# Idarubicinol myelotoxicity: a comparison of in vitro data with clinical outcome in patients treated with high-dose idarubicin

**DOI:** 10.1054/bjoc.1999.0957

**Published:** 2000-01-18

**Authors:** C Corsini, M Ghielmini, P Mancuso, F Tealdo, M Paolucci, M Zucchetti, P F Ferrucci, E Cocorocchio, M Mezzetti, A Mori, M Riggi, M D'Incalci, G Martinelli

**Affiliations:** Hemato/Oncology Department, European Institute of Oncology, Via Ripamonti 435, Milan, 20141, Italy; Oncology Department, Ospedale S. Giovanni, Bellinzona, Switzerland; Oncology Department, Mario Negri Institute, Via Eritrea 62, Milan, 20157, Italy; Epidemiology and Biostatistic Department, European Institute of Oncology, Via Ripamonti 435, Milan, 20141, Italy; Oncology Department, Pharmacia and Upjohn, Via Cook 1/2, Milan, 20152, Italy

**Keywords:** high-dose chemotherapy, idarubicin, idarubicinol, CFU-GM growth inhibition, haematotoxicity

## Abstract

We evaluated in vitro the toxicity of idarubicin and its active metabolite idarubicinol on haematopoietic progenitors, using human umbilical cord blood and peripheral blood progenitors to obtain dose–response curves. We treated 16 patients with poor prognosis lymphoma in a phase I–II trial of high-dose idarubicin and melphalan and investigated if idarubicinol persisting in patients' plasma at the time of transplantation (day 0), on day +1 and +2 could result in an inhibition of infused progenitors. Colony inhibition was correlated with pharmacokinetic data and with the time of patients' engraftment. Plasma samples obtained before idarubicin treatment demonstrated a colony-stimulating effect, increasing the cloning efficiency by 72%. The inhibitory activity on colony forming unit granulocyte–macrophage (CFU-GM) of patients' plasma collected on the day of transplantation was lower than expected from dose–response curves (21% measured vs 70% expected). The time to patients' WBC and PLT recovery correlated with the amount of CD34+ cells reinfused and, to a lesser extent, with the colony-inhibiting effect of patients' plasma. The correlation between idarubicinol concentration and CFU-GM inhibition was not significant. These data suggest that plasma drug concentration on the day of stem cell reinfusion may overestimate the toxicity of residual anthracyclines to the transplanted cells. © 2000 Cancer Research Campaign


British Journal of Cancer (2000) 82(3), 524–528
© 2000 Cancer Research Campaign
DOI: 10.1054/ bjoc.1999.0957, available online at http://www.idealibrary.com on IDEAL®

### Idarubicinol myelotoxicity: a comparison of in vitro data with clinical outcome in patients treated with high-dose idarubicin

**C Corsini[1], M Ghielmini[2], P Mancuso[1], F Tealdo[1], M Paolucci[1], M Zucchetti[3], PF Ferrucci[1], E Cocorocchio[1], M Mezzetti[4], A Mori[5], M Riggi[5], M D'Incalci[3] and G Martinelli[1]**

[1]Hemato/Oncology Department, European Institute of Oncology, Via Ripamonti 435, 20141 Milan, Italy; [2]Oncology Department, Ospedale S. Giovanni, Bellinzona, Switzerland; [3]Oncology Department, Mario Negri Institute, Via Eritrea 62, 20157 Milan, Italy; [4]Epidemiology and Biostatistic Department, European Institute of Oncology, Via Ripamonti 435, 20141 Milan, Italy; [5]Oncology Department, Pharmacia and Upjohn, Via Cook 1/2, 20152 Milan, Italy.

**Summary** We evaluated in vitro the toxicity of idarubicin and its active metabolite idarubicinol on haematopoietic progenitors, using human umbilical cord blood and peripheral blood progenitors to obtain dose–response curves. We treated 16 patients with poor prognosis lymphoma in a phase I–II trial of high-dose idarubicin and melphalan and investigated if idarubicinol persisting in patients' plasma at the time of transplantation (day 0), on day +1 and +2 could result in an inhibition of infused progenitors. Colony inhibition was correlated with pharmacokinetic data and with the time of patients' engraftment. Plasma samples obtained before idarubicin treatment demonstrated a colony-stimulating effect, increasing the cloning efficiency by 72%. The inhibitory activity on colony forming unit granulocyte–macrophage (CFU-GM) of patients' plasma collected on the day of transplantation was lower than expected from dose–response curves (21% measured vs 70% expected). The time to patients' WBC and PLT recovery correlated with the amount of CD34+ cells reinfused and, to a lesser extent, with the colony-inhibiting effect of patients' plasma. The correlation between idarubicinol concentration and CFU-GM inhibition was not significant. These data suggest that plasma drug concentration on the day of stem cell reinfusion may overestimate the toxicity of residual anthracyclines to the transplanted cells. © 2000 Cancer Research Campaign

**Keywords:** high-dose chemotherapy; idarubicin; idarubicinol; CFU-GM growth inhibition; haematotoxicity

Idarubicin (IDR) is a daunorubicin derivative frequently used in the treatment of haematological malignancies. Like other anthracyclines, IDR exerts its action by intercalating DNA and inhibiting topoisomerase II (Liu et al, 1983). In vitro studies and in vivo preclinical models showed IDR to be more cytotoxic than daunorubicin or doxorubicin (Salmon et al, 1981; Broggini et al, 1984; Dodion et al, 1987; Schott et al, 1989). Idarubicinol (IDRol), an alcohol metabolite present in large amounts in plasma after administration of IDR, is similarly cytotoxic in vitro as the parent compound, in contrast with other anthracyclines which metabolites have only a low cytotoxic effect. This peculiar characteristic of IDRol must probably be ascribed to its lipophilicity, which allows easier penetration into the cell (Kuffel et al, 1992).

The first evidence of IDR clinical activity was established in the treatment of patients with relapsed or refractory acute myelogenous leukemia (Vogler et al, 1992). More recently, clinical trials have demonstrated its activity in other malignancies such as the myelodysplastic syndromes (Greenberg et al, 1993), multiple myeloma (Chisesi et al, 1988) and advanced breast cancer (Twelves et al, 1995). Furthermore, IDR induced a high response rate (43%) in patients with relapsed or refractory intermediate–high-grade non-Hodgkin's lymphoma, with more than 10-months median duration of response (Case et al, 1993). In combination with other chemotherapeutic agents, IDR achieved response rates ranging from 47% to 80% with 7–12 months median duration of response (Dufour et al, 1993; Engert et al, 1995; Garay et al, 1997). We therefore decided to substitute mitoxantrone with IDR for patients with lymphoma undergoing high-dose chemotherapy and autologous stem cell transplantation according to the high-dose sequential protocol developed by Gianni et al (1993). In this scheme, high-dose IDR was associated with high-dose melphalan as conditioning regimen before autologous haematopoietic stem cell transplantation. The addition of anthracyclines to the conditioning regimen for bone marrow transplantation is associated with severe oral mucositis and slower haematopoietic recovery. These side-effects seem to be schedule-dependent and can be partially avoided when IDR is given as a continuous infusion between days –12 and –11 prior to bone marrow transplantation compared with days –7 and –1. Moreover, it was hypothesized that delayed engraftment may be due to the presence of IDR and its active metabolite IDRol at the time of the graft infusion and during the early post-transplant days, because of the very long half-life of these substances (Van Der Lely et al, 1989; Muus et al, 1993). Pharmacokinetic studies indicate in fact that the half-life of IDR and IDRol after oral administration ranges between 5 and 39 h and between 13 and 64 h respectively with a median value of 13.7 and 45.8 h. Similarly, median values of 16.2 h for IDR and of 54.4 h for IDRol were observed after intravenous administration (Camaggi et al, 1992; Robert et al, 1993).

It is believed that reinfusion of stem cells should be delayed until plasma concentrations of the drug and its active metabolite

*Received 10 March 1999*
*Revised 11 August 1999*
*Accepted 14 September 1999*

Correspondence to: C Corsini are below levels which are toxic to haematopoietic progenitor cells, but to date there are no data comparing IDRol plasma concentration to haematopoietic stem cell toxicity. We therefore planned to study IDR and IDRol cytotoxicity first on clonogeneic cells derived from human umbilical cord blood (HCB), then on peripheral blood progenitor cells (PBPC) from mobilized patients. Furthermore, we evaluated the inhibitory activity on CFUs growth of plasma samples collected from IDR treated patients at the time of transplantation and we correlated this inhibitory capacity with the time to engraftment.

### PATIENTS AND METHODS

### Cells

HCB samples were obtained after informed consent of the mother from normal vaginal deliveries. In all cases 40–50 ml of HCB were collected in sterile heparin-containing tubes, stored at +4°C and processed within 24 h. Mononuclear cells (MNC) were separated using a Ficoll gradient and depleted of adherent cells by overnight incubation in IMDM–20% fetal bovine serum (FBS) at 37°C, 5% carbon dioxide. Cells were cryopreserved in aliquots and stored in liquid nitrogen until use. In order to verify that HCB drug sensitivity can be predictive of the PBPC sensitivity, we tested MNC obtained from leukapheresis products (AP). AP mononuclear cells were separated by density centrifugation, depleted of adherent cells, cryopreserved and stored as described above.

### Cytotoxic treatment of haematopoietic cells

We tested IDR, IDRol and doxorubicin as a reference compound. All drugs, provided by Pharmacia and Upjohn (Milan, Italy), were reconstituted in sterile water and the concentration of the solutions were verified by high-performance liquid chromatography (HPLC). Aliquoted drugs were stored at –80°C until use. After thawing, HCB and AP mononuclear cells were incubated for 1 h or 24 h at 37°C in a 1 ml final volume of serum-free IMDM containing the drug at the concentration to be tested. After washing twice with IMDM–10% FBS, cells were plated in clonogeneic assays. For each drug, a first set of four experiments was set up at concentrations ranging over five logs; in a second set of experiments doses were chosen within the $ID_{10}$–$ID_{90}$ range (range of concentrations inhibiting the growth of 10–90% of progenitors) to better evaluate the dose–response curve: these ranges were between 50 and 200 ng ml$^{-1}$ for 1-h exposure and between 5 and 20 ng ml$^{-1}$ for 24-h exposure to IDR and its metabolite, between 250 and 2000 ng ml$^{-1}$ for 1-h exposure and between 25 and 200 ng ml$^{-1}$ for 24-h exposure to doxorubicin.

We investigated the inhibitory effect of IDRol present in patients' plasma samples by incubating HCB mononuclear cells (all from the same donor) for 24 h with 1 ml of serum-free IMDM or 1 ml of undiluted plasma obtained from patients before IDR infusion and 96, 120 and 144 h after PBPC infusion (corresponding to day of the transplant, day +1 and +2).

We also investigated IDRol cytotoxicity when HCB mononuclear cells were incubated for 24 h in IMDM supplemented with 40 mg ml$^{-1}$ (mean normal serum level) human serum albumin. In this case, the medium containing the anthracycline and the albumin was preincubated without cells at 37°C for 15 min to allow the binding of the drug.

### Clonogeneic assay

Clonogeneic potential was assessed in a semisolid culture system using a commercially available methylcellulose-based medium containing 30% FBS, 3 U ml$^{-1}$ recombinant human (rhu) erythropoietin, 50 ng ml$^{-1}$ rhu stem cell factor, 10 ng ml$^{-1}$ rhu granulocyte–macrophage colony stimulating factor (GM-CSF) and 10 ng ml$^{-1}$ rhu interleukin-3 (Stem Cell Technologies, Vancouver, Canada). Cells were plated in duplicate and incubated for 14 days in a fully humidified atmosphere with 5% carbon dioxide at 37°C. Colonies consisting of more than 50 cells were scored at day 14 under an inverted microscope and classified as CFU-GM or BFU-E according to previously described criteria (Coutinho et al, 1993). Only experiments with a minimal cloning efficiency of 30 CFU-GM per $5 \times 10^4$ adherent-cell-depleted MNC were considered.

### Patients and treatment

Sixteen patients with poor prognosis Hodgkin's or non-Hodgkin's lymphoma were enrolled in a phase I–II trial of IDR dose-escalation in combination with high-dose melphalan. The median age was 36 years (range 19–62). Two patients had relapsed Hodgkin's disease and 14 had non-Hodgkin's lymphoma; five received autologous transplantation for relapse and 11 as consolidation after first-line chemotherapy.

PBPC were collected after mobilization with cyclophosphamide 7 g m$^{-2}$ followed by G-CSF 5 μg kg$^{-1}$ day. After further cycles of chemotherapy, the myeloablative regimen was administered: IDR was given intravenously as a continuous infusion at escalating dosages from 12 to 17 mg m$^{-2}$ on days –6, –5, –4 before transplant, followed by melphalan 180 mg m$^{-2}$ on day –2. The graft, given on day 0, contained a median of $3.4 \times 10^6$ CD34+ cells kg$^{-1}$ (range 2.1–9.6). All patients subsequently received G-CSF 5 mg kg$^{-1}$ day from day +5 until neutrophils reached $\geq 1 \times 10^9$ l$^{-1}$ for 3 consecutive days.

### Plasma samples and pharmacokinetics

Blood samples were drawn during and after IDR administration. Ten millilitres of heparinized blood were drawn 1 h before the start, then 1, 3, 6, 12, 24, 36, 48, 72 h after the start of IDR infusion and 0.5, 1, 3, 6, 12, 24, 36, 48, 72, 96, 120, and 144 h after the end of infusion. Blood samples were centrifuged immediately at 1000 ***g*** for 10 min at +4°C. Plasma samples were collected and stored at –80°C until use. IDR and IDRol plasma concentrations were measured in each sample by HPLC.

### Analysis of data and statistics

Inhibition of colony formation was the proportion of the mean number of colonies growing from treated cells, compared to the mean colony number in the control. Dose–response curves were produced using a standard software program (SAF). The concentration inhibiting the growth of 70% of progenitors ($ID_{70}$) was calculated from graphical analysis (Figure 1). The $ID_{70}$ value was chosen to ensure accurate prediction of clinically important levels of drug exposure (Parchment et al, 1998).

Comparison of the growth inhibition of the various progenitors was performed by fitting a linear regression with logit of inhibition as a response variable and testing first the effect of HCB versus AP

*© 2000 Cancer Research Campaign* 

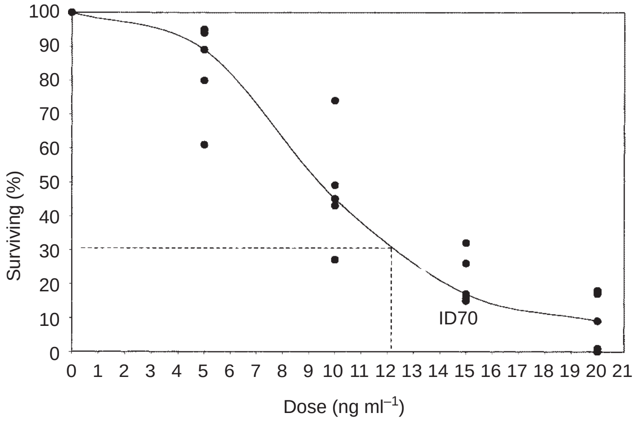

**Figure 1** Dose–response curve of AP cells on CFU-GM exposed for 24 h to IDRol. Median of 5 experiments (—) and individual data points. $ID_{70}$ represents the concentration at which 30% of colonies survive.

progenitors and subsequently the effect of CFU-GM versus BFU-E. The variability between subjects was taken into account. Correlations were calculated using the Pearson correlation coefficient ($r$). Probability values are obtained by transformation of correlation coefficient ($(n-2)^{1/2}r/(1-r^2)^{1/2}$) as $t-$Student with ($n-2$) degrees of freedom. Differences with a $P$-value < 0.05 were considered statistically significant.

### RESULTS

### In vitro cytotoxicity

The mean cloning efficiency (colonies per $5 \times 10^4$ adherent-cell-depleted MNC) of HCB and AP progenitors was comparable, being respectively 65 ± 23 and 108 ± 56 for CFU-GM, and 50 ± 24 and 99 ± 43 for BFU-E.

Growth inhibition was determined for all haematopoietic precursors assessable by this assay but the $ID_{70}$ was calculated only for CFU-GM and BFU-E since CFU-mix were scored in too low numbers to allow any analysis.

Cytotoxicity test results are reported in Tables 1 and 2. Progenitors from both sources demonstrated a similar sensitivity to the drugs, the differences not being statistically significant. IDRol showed a toxicity 2–2.5 times lower than IDR. For all drugs, prolonged exposure was 9–11 times more toxic than 1-h exposure.

The median $ID_{70}$ for CFU-GM and BFU-E after 24-h exposure to IDRol in IMDM supplemented with human serum albumin was 15 ng $ml^{-1}$ (range 9–18) and 12 ng $ml^{-1}$ (range 7–18) respectively. These values are not significantly different from the experiments performed incubating the cells in medium without albumin.

### Pharmacokinetic results

In all the 16 patients, IDR was completely cleared from plasma at the time of transplant, its median half-life being 18.8 h (range 8.9–58.8) ($n$ = 14). In contrast, IDRol levels ranged from 3.3 to 17.4 ng $ml^{-1}$ (median value 10.2) on the day of PBPC infusion, from 3.6 to 13 ng $ml^{-1}$ (median value 8.8) on day +1 and from 3.2 to 11.4 ng $ml^{-1}$ (median value 6.2) on day +2 (Table 3). IDRol half-life ranged between 42.5 and 90.2 h (median value 51.2 h, $n$ = 16).

### Cytotoxic activity of plasma samples obtained from IDR treated patients

The mean cloning efficiency of CFU-GM after 24 h exposure was higher for patients' plasma both at baseline (101 ± 27 colonies per $5 \times 10^4$ adherent-cell-depleted MNC) and on the day of transplantation (81 ± 20 colonies) compared to the same cells in serum-free IMDM (59 ± 15 colonies).

HCB mononuclear cells incubated for 24 h in patients' plasma drawn at day 0 showed a median CFU-GM growth inhibition of 21% (range 0–43). With plasma from day +1, the growth inhibition was still 20% (range 0–40) and on day +2 it fell to 11.5% (range 0–32) (Table 3). Statistical analysis revealed a weak, not significant correlation between IDRol plasma levels and the degree of in vitro cytotoxicity.

**Table 1** Median ID70 and range (ng $ml^{-1}$) of IDR, IDRol and the reference compound doxorubicin on HCB and AP after 1-h exposure

| | HCB ($n$ = 4) | | AP ($n$ = 5) | |
|---|---|---|---|---|
| | CFU-GM | BFU-E | CFU-GM | BFU-E |
| Idarubicin | 70 (55–105) | 60 (40–80) | 50 (45–60) | 45 (35–60) |
| Idarubicinol | 130 (80–170) | 120 (105–145) | 100 (85–160) | 85 (60–130) |
| Doxorubicin | 1300 (800–1700) | 1600 (900–2500) | 1300 (750–1600) | 1800 (750–2100) |

**Table 2** Median ID70 and range (ng $ml^{-1}$) of IDR, IDRol and the reference compound doxorubicin on HCB and AP after 24-h exposure

| | HCB ($n$ = 4) | | AP ($n$ = 5) | |
|---|---|---|---|---|
| | CFU-GM | BFU-E | CFU-GM | BFU-E |
| Idarubicin | 5 (3.5–8) | 6 (4–9) | 4.5 (2–6) | 4 (2–5.5) |
| Idarubicinol | 12 (7–17) | 11 (9–16) | 12 (9–16) | 10 (7–14) |
| Doxorubicin | 120 (90–160) | 160 (60–180) | 145 (80–190) | 160 (120–220) |

© 2000 Cancer Research Campaign

**Table 3** Patient's characteristics, IDRol plasma levels and CFU-GM growth inhibition

| Patient no. | Diagnosis | Dose of IDR ($mg\ m^{-2}$) | Activity at time of PBPC infusion | | Activity on day +1 | | Activity on day +2 | | CD34+ cells infused | Time to haematopoietic reconstitution (days) | |
|---|---|---|---|---|---|---|---|---|---|---|---|
| | | | ($ng\ ml^{-1}$) | Inhibition (%) | ($ng\ ml^{-1}$) | Inhibition (%) | ($ng\ ml^{-1}$) | Inhibition (%) | ($\times 10^6\ kg^{-1}$) | WBC > $1 \times 10^9\ l^{-1}$ | PLT > $10 \times 10^9\ l^{-1}$ |
| 1 | NHL | 12 | 3.3 | 0 | 3.6 | 4 | 3.2 | 0 | 3.7 | 15 | 16 |
| 2 | NHL | 12 | 8.4 | 25 | 6.5 | 21 | 4.6 | n.e.° | 5.6 | 10 | 11 |
| 3 | NHL | 14 | 6.6 | 14 | 6.4 | 0 | 3.8 | 0 | 2.1 | 13 | 13 |
| 4 | HD | 14 | 14.2 | 20 | 13 | n.e.[a] | 11.4 | n.e.° | 3.5 | 14 | 18 |
| 5 | NHL | 14 | 13.6 | 16 | 11.4 | 18 | 8 | 13 | 2.1 | 12 | 12 |
| 6 | NHL | 14 | 14.4 | 20 | 11.7 | 18 | 9.4 | 22 | 2.9 | 17 | 19 |
| 7 | NHL | 14 | 10.4 | 34 | 9.8 | 36 | 7.4 | 32 | 7.5 | 13 | 12 |
| 8 | NHL | 15 | 17.4 | 0 | 11.2 | 0 | 9.4 | 0 | 9.6 | 11 | 11 |
| 9 | HD | 15 | 13.6 | 43 | 6.5 | 40 | 5.4 | 27 | 2.1 | 15 | 15 |
| 10 | NHL | 16 | 9.2 | 18 | 10.4 | 20 | 7.8 | 14 | 8.2 | 14 | 14 |
| 11 | NHL | 16 | 17.3 | 26 | 12.2 | 22 | 9.4 | 12 | 8.1 | 12 | 13 |
| 12 | NHL | 16 | 11.9 | 40 | 9.9 | 28 | 6.4 | 7 | 3.4 | 13 | n.e. |
| 13 | NHL | 17 | 10.1 | 33 | 5.4 | 25 | 5 | 10 | 3.3 | 16 | 16 |
| 14 | NHL | 17 | 7.5 | 12 | 5.2 | 5 | 3.8 | 0 | 4.3 | 13 | 13 |
| 15 | NHL | 17 | 8.2 | 22 | 7.9 | 15 | 5.1 | 11 | 2.4 | 19 | 38 |
| 16 | NHL | 17 | 9.6 | 37 | 7.5 | 24 | 6.1 | 16 | 3.1 | 16 | 18 |
| Median | | | 10.25 | 21 | 8.85 | 20 | 6.25 | 11.5 | 3.45 | 13.5 | 14 |

[a] Inhibition not evaluable, but reasonably ≤ 20%.

### Haematopoietic reconstitution

Table 3 shows the pattern of haematopoietic reconstitution for each patient. All patients achieved a sustained and rapid haematopoietic recovery. They reached $1 \times 10^9$ WBC $l^{-1}$ and $10 \times 10^9$ PLT $l^{-1}$ in a median time of 13.5 days (range 10–19) and 14 days (range 11–38) respectively. A significant negative correlation ($P < 0.05$) was found between the dose of CD34+ cells $kg^{-1}$ infused and the median time to $1 \times 10^9$ WBC $l^{-1}$ and $10 \times 10^9$ PLT $l^{-1}$. Only a weak, not significant correlation could be found between IDRol plasma concentration on the day of PBPC infusion, the grade of colony inhibition and the haematopoietic engraftment. A significant correlation ($P < 0.05$) was found between the level of toxicity observed on CFU-GM with plasma samples drawn on day +1 and +2 and the time to WBC and PLT recovery.

### DISCUSSION

Van Der Lely et al (1989) and Muus et al (1993) reported that the addition of anthracyclines to a conditioning regimen for bone marrow transplantation may be associated with a slower haematopoietic recovery. With this study we wanted to obtain more information on the toxicity of IDR and IDRol on haematopoietic progenitors and to investigate if residual IDRol persisting in patients' plasma at the time of transplantation, could result in an inhibition of transplanted PBPC. We also aimed at correlating this plasma inhibition potential with the time of engraftment.

To obtain data on the sensitivity of haematopoietic progenitors, we first performed in vitro myelotoxicity experiments testing IDR, IDRol, and doxorubicin as a reference compound. We used HCB as a source of haematopoietic progenitors as suggested by Leglise et al (1996) and Ghielmini et al (1997, 1998). The experiments with doxorubicin produced results similar to the published ones (Minderman et al, 1994; Ghielmini et al, 1998). With IDR and its alcohol metabolite we found a slightly smaller toxicity compared to reported data (Minderman et al, 1994; Dodion et al, 1997), but these discrepancies can be explained by different times of exposure and culture conditions. On the other hand the proportion of cytotoxicity between IDR and IDRol we have observed (IDRol 2–2.5 times less toxic than IDR) agrees with the published data (Dodion et al, 1987; Minderman et al, 1994). We demonstrated that the sensitivity of HCB to anthracyclines is similar to that of PBPC. To our knowledge, only few groups have been using AP as a substrate for haematotoxicology studies; further experimental protocols testing other classes of drugs are therefore required to validate PBPC as a cellular model for haematotoxicology.

Because it was reported that the pattern of toxicity of a drug on haematopoietic cells in vitro could predict the pattern of myelotoxicity in vivo (Gribaldo et al, 1996; Parchment et al, 1998), we compared the cytotoxic activity of IDRol-containing plasma samples to previously constructed IDRol dose–response curves and correlated it with pharmacokinetic data and time of engraftment. In accordance with data from other authors (Camaggi et al, 1992; Robert et al, 1993), pharmacokinetic studies on our cohort indicated that IDRol persists in plasma for a prolonged time after IDR administration and it is still present at the time of haematopoietic progenitors transplant. Plasma samples showed a median colony inhibition of 21% on the day of transplantation and lower values in the subsequent 2 days. In contrast, the median IDRol concentration detected in plasma at the time of PBPC infusion (10.25 ng $ml^{-1}$) was able to inhibit the growth of 70% of CFU-GM progenitors ($ID_{70}$) in the 24-h in vitro exposure. Despite that, all patients achieved a rapid and sustained haematopoietic recovery: no delay or failure of engraftment occurred. Only a weak correlation was found between IDRol plasma concentration on the day of PBPC infusion, the grade of in vitro cytotoxicity and the haematopoietic engraftment. In vitro assessment of IDRol myelotoxicity appears not to reflect the situation in vivo and the clinical relevance of persisting IDRol at the time of stem cell reinfusion remains unclear.

We observed an important variability among patients concerning IDRol plasma levels, the cytotoxic potential of plasma samples and the clinical outcome. We supposed that the variability in the plasma inhibition activity might be related to differences in the levels of free IDRol among plasma samples since IDRol is extensively bound (about 94%) to plasma proteins in vivo (Camaggi et al, 1992). Nevertheless, we found no evidence that albumin influence in vitro IDRol cytotoxicity, but the possible effect of binding to other plasma proteins was not tested.

*© 2000 Cancer Research Campaign*

Other factors not present in our in vitro system may play an important role in determining the biological activity of the drug and explaining the lack of in vitro–in vivo correlation: one is the observed stimulatory activity of patients' plasma on HCB progenitors, possibly due to endogenous growth factors exerting a stimulating and protective effect on reinfused haematopoietic progenitors. Another additional factor present in vivo is the marrow stromal environment which is crucial in supporting and regulating the proliferation and differentiation of haematopoietic progenitors: the extracellular matrix can modulate haematopoietic cells adhesion and bind regulatory molecules, and the stromal cells are capable of producing growth factors (Campbell et al, 1988). Such interaction with the stromal environment may play a role in determining the in vivo stem cells chemosensitivity.

Taken together, our data indicate that biological and clinical factors other than the direct toxicity of the drug must be considered in evaluating IDRol haematotoxicity in vivo. Consequently, caution must be paid in extrapolating in vitro data to the clinical situation and further studies are required to better understand the predictive value of haematotoxicity tests. Additionally, it appears that plasma drug concentrations on the day of PBPC reinfusion may be less important than previously believed, in determining the speed of haematological recovery after stem cell transplantation.

### ACKNOWLEDGEMENTS

We thank Dr G Parma and the midwives of the obstetric-gynaecology department of Ospedale di Monza (Monza, Italy) for their help in collecting cord blood.

### REFERENCES

Broggini M, Italia C, Colombo T, Marmonti L and Donelli MG (1984) Activity and distribution of i.v. and oral 4-demethoxydaunorubicin in murine experimental tumours. *Cancer Treat Rep* **68**: 739–747

Camaggi CM, Strocchi E, Carisi P, Martoni A, Tononi A, Guaraldi M, Strolin-Benedetti M, Efthymiopoulos C and Pannutti F (1992) Idarubicin metabolism and pharmacokinetic after intravenous and oral administration in cancer patients. A cross-over study. *Cancer Chemother Pharmacol* **30**: 307–316

Campbell AD and Wicha MS (1988) Extracellular matrix and the haematopoietic microenvironment. *J Lab Clin Med* **112**: 140–146

Case DC, Gerber MC, Gams RA, Crawford J, Votaw ML, Higano CS, Pruitt BT and Gould J (1993) Phase II study of intravenous idarubicin in unfavourable non-Hodgkin's lymphoma. *Leuk Lymphoma* **10**: 73–79

Chisesi T, Capnist G, De Dominicis E and Dini E (1988) A phase II study of idarubicin (4-demethoxydaunorubicin) in advanced myeloma. *Eur J Cancer Clin Oncol* **24**: 681–684

Coutinho LH, Gilleece MH, De Winter EA, Will A and Testa NG (1993) Clonal and long-term cultures using human bone marrow. In: *Haemopoiesis: a Practical Approach*, Testa NG, Molineux G (eds), pp. 75–105. Oxford University Press: New York

Dodion P, Sanders C, Rombaut W, Mattelaer MA, Rozencweig M, Styckmans P and Kenis Y (1987) Effect of daunorubicin, carminomycin, idarubicin and 4-demethoxydaunorubicinol against normal myeloid stem cells and human malignant cells in vitro. *Europ J Cancer Clin Oncol* **23**: 1909–1914

Dufour P, Mors R and Lamy T (1993) Idarubicin (IDA) and high-dose aracytine (HD-AraC): a new promising salvage treatment in relapsed or refractory non Hodgkin's lymphoma. *Proc Am Soc Clin Oncol* **12**: 364 (abstract 1229)

Engert A (1995) Treatment of large cell lymphoma in second relapse. Lymphoma the next question. October 19–21, San Antonio. TX (abstract)

Garay G, Dupont J, Dragosky M, Nucifora E, Cacchione R, Schnidring P, Fernandez J, Abel-Alzueta A, Riveros D, Noviello V, Beguelin R, Campestri R, Albera C, Nicastro M and Triguboff E (1997) Combination salvage chemotherapy with MIZE (mesna-ifosfamide, idarubicin and etoposide) for relapsing or refractory lymphoma. *Leuk Lymphoma* **26**: 595–602

Ghielmini M, Bosshard G, Capolongo L, Geroni MC, Pesenti E, Torri V, D'Incalci M, Cavalli F and Sessa C (1997) Estimation of the haematological toxicity of minor groove alkylators using tests on human cord blood cells. *Br J Cancer* **75**: 878–883

Ghielmini M, Colli E, Bosshard G, Pennella G, Geroni C, Torri W, D'Incalci M, Cavalli F and Sessa C (1998) Hematotoxicity on human bone marrow- and umbilical cord blood-derived progenitor cells and in vitro therapeutic index of methoxymorpholinyldoxorubicin and its metabolite. *Cancer Chemoth Pharmacol* **42**: 235–240

Gianni AM, Siena S, Bregni M, Lombardi F, Gandola L, Di Nicola M, Magni M, Peccatori F, Valagussa P and Bonadonna G (1993) High-dose sequential chemotherapy with peripheral blood progenitor cell support for relapsed or refractory Hodgkin's disease. A 6-year update. *Ann Oncol* **4**: 889–891

Greenberg BR, Reynolds RD, Charron CB, Squillace KM, Lessins LS, Case DC and Gams RA (1993) Treatment of myelodysplastic syndrome with daily oral idarubicin. *Cancer* **71**: 1989–1992

Gribaldo L, Bueren J and Deldar A (1996) The use of in vitro systems for evaluating haematotoxicity. *Atla* **24**: 211–231

Kuffel M, Reid JM and James MM (1992) Anthracyclines and their C-13 alcohol metabolites. Growth inhibition and DNA damage following incubation with human tumor cells in culture. *Cancer Chemother Pharmacol* **30**: 51–57

Leglise MC, Darodes de Taily P, Vignot JL, Le Bot MA, Le Roux AM and Richè C (1996) A cellular model for drug interactions in hematopoiesis: the use of human umbilical cord blood progenitors as a model for the study of drug-related myelosuppression of normal hematopoiesis. *Cell Biol Toxicol* **12**: 39–53

Liu LF, Rowe TC, Yang L, Tekey KM and Chen GL (1983) Cleavage of DNA by mammalian DNA topoisomerase II. *J Biol Chem* **258**: 15365–15370

Minderman H, Linssen P, Van der Lely N, Wessels J, Boezeman J, De Witte F and Haanen C (1994) Toxicity of idarubicin and doxorubicin towards normal and leukemic human bone marrow progenitors in relation to their proliferative status. *Leukemia* **8**: 382–387

Muus P, Donnelly P, Schattenberg A, Linssen P, Minderman H, Dompeling E and De Witte T (1993) Idarubicin-related side effects in recipients of T-cell-depleted allogeneic bone marrow transplants are schedule dependent. *Semin Oncol* **20**: 47–52.

Parchment RE, Gordon M, Grieshaber CK, Sessa C, Volpe D and Ghielmini M (1998) Predicting hematological toxicity (myelosuppression) of cytotoxic drug therapy from in vitro tests. *Ann Oncol* **9**: 357–364

Robert J (1993) Clinical pharmacokinetics of idarubicin. *Clin Pharmacokinet* **24**: 275–288

Salmon SE, Liu RM and Casazza AM (1981) Evaluation of new anthracycline analogs with the human tumor stem cell assay. *Cancer Chemother Pharmacol* **6**: 103–110

Schott B and Robert J (1989) Comparative cytotoxicity, DNA synthesis inhibition and drug incorporation of eight anthracyclines in a model of doxorubicin-sensitive and -resistant rat glioblastoma cells. *Biochem Pharmacol* **38**: 167–172

Twelves CJ (1995) Oral idarubicin in solid tumors chemotherapy. *Clin Drug Invest* **9** (Suppl. 2): 39–54

Van Der Lely N, De Witte T, Raemaekers J, Schattenberg A and Haanen C (1989) Anthracyclines added to the conditioning regimen for allogeneic bone marrow transplantation are associated with a slower haematopoietic recovery. *Bone Marrow Transplant* **4**: 163–166

Vogler WR, Velez-Garcia E, Weiner RS, Flaum MA, Bartolucci AA, Omura GA, Gerber MC and Banks PL (1992) A phase III trial comparing idarubicin and daunorubicin in combination with cytarabine in acute myelogenous leukemia: a Southeastern Cancer Study Group study. *J Clin Oncol* **10**: 1103–1111

© 2000 Cancer Research Campaign
